# Probiotic, antimicrobial and anticancer properties of *Lysinibacillus macroides*, *Kurthia huakuii*, and *Enterococcus faecium* isolated from freshwater snail gut microbiota

**DOI:** 10.1186/s12896-025-01038-5

**Published:** 2025-09-03

**Authors:** Reham Alaa Eldin Shaker, Rasha A. Hashem, Mariam Hassan, Amina M. Ibrahim, Yasser M. Ragab, Rania Abdelmonem Khattab

**Affiliations:** 1 Department of Microbiology and Immunology, Faculty of Pharmacy, Cairo University, Kasr Al-Aini, Cairo, 11562 Egypt; 2 Department of Microbiology and Immunology, Faculty of Pharmacy, Galala University, New Galala City, Suez 43511 Egypt; 3 Department of Medical Malacology, Theodor Bilharz Research Institute, Corniche El-Nile St., Imbaba, Giza, 12411 Egypt; 4 Department of Microbiology and Immunology, Faculty of Pharmacy, Sinai University (EL-Arish Branch), Sinai, 16020 Egypt

**Keywords:** Probiotics, Freshwater snails, *Enterococcus faecium*, Anticancer activity, Microbiota, Immunohistochemistry

## Abstract

**Background:**

The composition and roles of intestinal microbial populations have been clarified including mammals and humans however, less is understood concerning the gut microbiota of mollusks. For the first time, we investigated non-parasite transmitting freshwater snails *Lanistes carinatus* (*L. carinatus*), *Cleopatra bulimoides* (*C. bulimoides*) and *Helisoma duryi* (*H. duryi*) gut microbiota as a source of probiotic strains with anticancer potential and explore their microbial population structure.

**Results:**

Our investigation demonstrated significant variation in microbial richness, identifying 32 bacterial phyla across the three snail species. *Pseudomonadota* (44–60%) and *Bacteroidota* (17–20%) were identified as the predominant phyla in all snails, with *p* value = 0.28 and 0.39, respectively in relative abundance. Distinct compositional changes were observed as *L. carinatus* had a greater abundance of *Bacillota*. *H. duryi* exhibited higher microbial diversity with *Verrucomicrobiota* and *Cyanobacteria* comprising 5–20% of its gut microbiota. *Lysinibacillus macroides* (*L. macroides*), *Kurthia huakuii* (*K. huakuii*) and *Enterococcus faecium* (*E. faecium*) were isolated from *L. carinatus*, *C. bulimoides* and *H. duryi*, respectively. *L. macroides*, *K. huakuii* and *E. faecium* demonstrated antimicrobial efficacy towards selected pathogenic strains. The bacterial isolates displayed significant tolerance to acidic pH and bile salts concentrations (0.3% and 0.7% w/v). The cytotoxicity of the microbial isolates secreted metabolites was examined using the MTT assay. Cytopathological changes and caspase-3 / TNF α immunohistochemistry were examined on Caco-2 cells. Results demonstrated the anticancer activity of the metabolites of the three microbial isolates on Caco2 cells where *K. huakuii* exhibited the highest enhancement in apoptosis and necrosis.

**Conclusions:**

Our study identified diverse bacterial populations in freshwater snail gut microbiota with compositional differences. The isolated bacterial strains showed promising antimicrobial properties and anticancer potential, particularly *K. huakuii*. These results suggest that snails could be used as niche sources for beneficial bacteria with biotechnological and therapeutic applications.

**Graphical Abstract:**

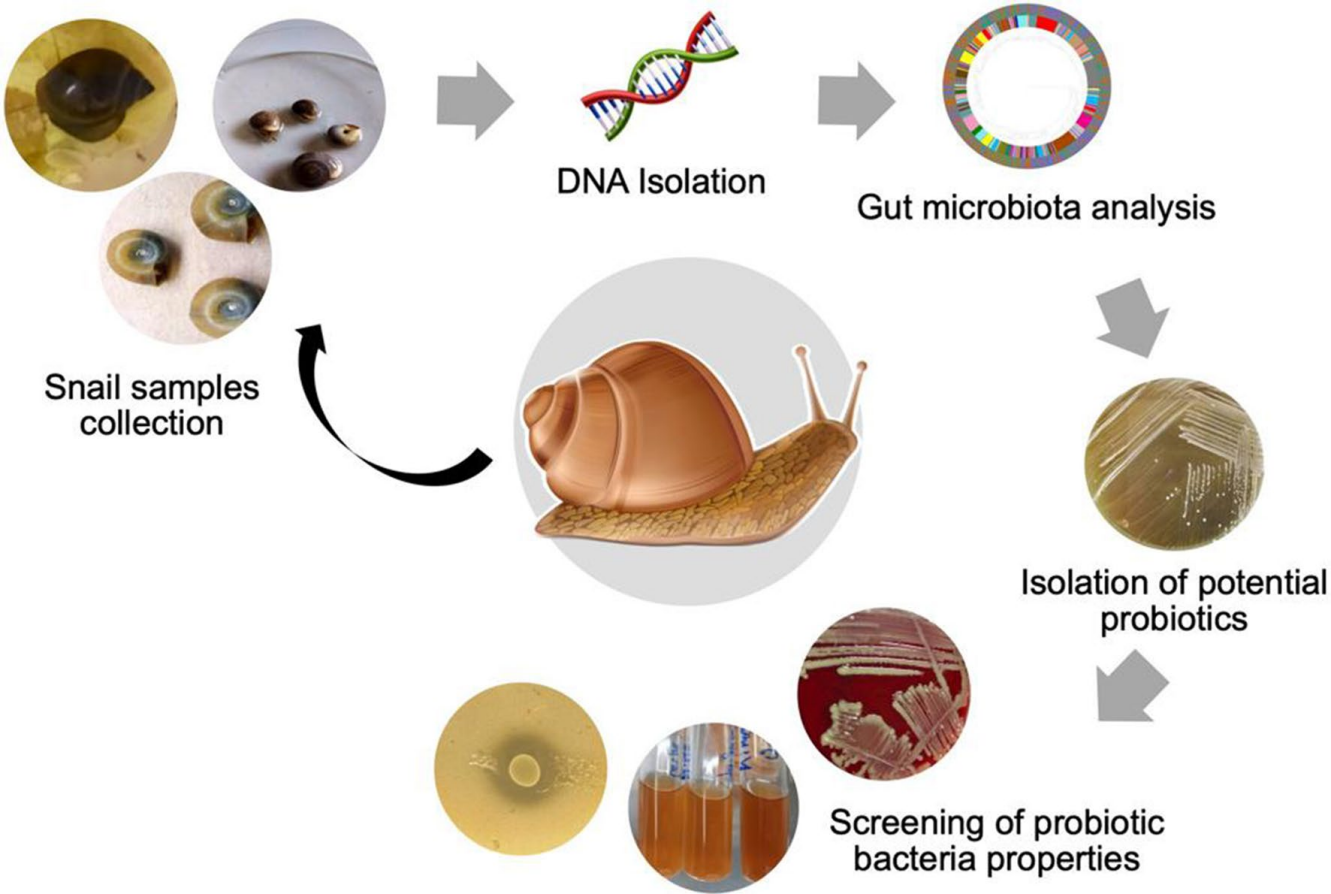

**Supplementary Information:**

The online version contains supplementary material available at 10.1186/s12896-025-01038-5.

## Background

The search for new microbial sources that might have potential medicinal and biotechnological uses is becoming crucial requirement due to the increasing worldwide problems of antibiotic resistance and environmental pollution and as traditional microbial reservoirs such as soil, marine environments, and mammalian microbiomes have been extensively studied [1–3]. The unique ecological niche of the snail gut may harbor novel and distinct strains that are not commonly found in other sources with various properties and possible new applications [4]. Several marine mollusks have been reported as sources of potent anticancer peptides [5, 6] emphasizing the therapeutic potential of mollusk-derived bioactive compounds.

Fresh water snails possess a unique gastrointestinal tract that harbors diverse microbial communities [4]. In addition to its role in digesting, the gut microbiota of aquatic snail species impacts the host’s nutrition, development, reproduction, immunology, and susceptibility to infection [7]. The presence of antibacterial and antifungal capabilities in snail mucus promotes the potential that the bacteria in snails’ gut microbiota may develop antimicrobial compounds [8].

Furthermore, according to Charrier et al. [9] and Pinheiro et al. [10] important biological processes including cellulose breakdown and immunological boosting have been linked to the gut microbiota of gastropods. *L. carinatus*,* C. bulimoides* and *H. duryi* are non-transmitting parasites, freshwater snails found throughout Africa. The African apple snail, scientifically known as *L. carinatus* have an important role in ecosystems. It serves as a measure of metal contamination and aids in assessing environmental conditions [11]. The ability of these freshwater snails to resist parasites that are associated with their gut microbiota could provide important clues for improving human health [12].

The recognition of probiotics has expanded significantly in recent decades for their capability to improve our health. These beneficial bacteria are known to boost the immune system, enhance digestion and support well-being [13]. Probiotics have long been associated with fermented meat and dairy products, but recent studies have shown that they may also be found in niche places, like snails [14–16]. Recent studies reported probiotics isolated from freshwater snails with biological activities [16, 17]. Extensive studies have investigated how probiotics may alleviate a variety of health problems [18–21]. Each probiotic strain offers benefits by influencing biological pathways such as maintaining a healthy gut microbiota balance, enhance intestinal barrier function, produce antimicrobial agents, and modulate immune responses [22]. Probiotic microorganisms can be added to nutritional supplements, capsules, or functional foods for more beneficial effect [15]. This work’s fundamental aim is to explore the gut microbiota of *L. carinatus*,* C. bulimoides*, and *H. duryi* to discover beneficial bacteria as probiotics and evaluate their functional potential.

## Methods

### Collection of snails and isolation of potential probiotic bacterial strains

Thirty non-parasite transmitting adult freshwater snails’ samples *L. carinatus*, *C. bulimoides* and *H. duryi* were attained from their natural habitat in different parts of Egypt (El Qalyubia and Giza governorate). The snail’s samples were gathered, surface sterilized and kept in a sterile container. Briefly, we collected fresh fecal samples (70 mg) aseptically from thirty specimens of each snail species and immediately inoculated in 10 ml of De Man–Rogosa–Sharpe (MRS) broth (Biolife, Spain) at 37 °C for 48 h. A volume of 100 µl of MRS broth was evenly distributed on the surface of MRS agar and placed in a 37 °C incubator with 5% CO_2_ for 48 h. Following incubation, colonies with diverse morphology were chosen arbitrarily and sub cultured on MRS agar plates to obtain pure bacterial colonies. Isolates were preserved as stocks in MRS broth at − 80 °C in 25% glycerol according to Adetoye et al. [23].

### Molecular identification of probiotic bacteria candidate

As per the manufacturer’s manual, the DNA was purified from the bacterial colonies utilizing DNA Purification Kit (Promega, USA). PCR was carried out to amplify the 16S rRNA gene amplicons employing primer pairs 1492r (5’GGT TAC CTT GTT ACG ACT T 3’) and 8f (5′AGA GTT TGA TCC TGG CTC AG 3’) [24]. The PCR was executed through an initial denaturation step of 95 °C for 2 min, followed by a second denaturation for 15 s, annealing of 53 °C for 20 s, and an extension of 72 °C for 90 s with a final extension step for one min at 72 °C repeated for 30 cycles. Thermal cycler (Boeco, USA) was used for this purpose in conjunction with COSMO PCR Red Master Mix from Willow Fort (UK). FavorPrep GEL/PCR Purification Mini Kit (Favrogen, Taiwan) was used for the purification procedure of the PCR products and sequenced at Macrogen (USA). Sequences were referenced with the existing sequences of DNA utilizing the BLASTN database at the National Center for Biotechnology Information (NCBI).

### Gut microbiota analysis

Collected snails were grouped into three groups each consisting of 10 snails. Following the directions provided by the manufacturer, we used a QIAamp Fast DNA Stool Mini Kit (Qiagen, Germany) to purify the DNA of the snails’ gut microbiota. The purified DNA was preserved at − 80 ◦C. A Nanodrop spectrophotometer (IMPLEN, Germany) was used to determine the quantity and purity of the extracted DNA. For verification of the quality of the DNA purified an aliquot was run down on agarose gel electrophoresis, confirming the existence of high molecular weight DNA bands. 16S rDNA amplicons were generated via amplification of V3–V4 region using paired primers 341F 5’-CCTACGGGNGGCWGCAG-3’ and 805R 5’– GACTACHVGGGTATCTAATCC- 3’ [25]. Amplicons with 300 bp length and pair end sequences were acquired through Illumina MiSeq sequencing system (Illumina, California, USA). The raw sequences data acquired were evaluated by Qiime 2 [26]. The demultiplexed data were denoised and trimmed to remove primers and barcodes through DADA2 [27]. Sequences were classified with pre-trained Naïve Bayes classifiers (Greengenes 2v.132 99%) [28]. Rarefaction curves, alpha and beta diversity indices were generated using Qiime2 software. Furthermore, using microbiome analyst principal component analysis (PCoA) of weighted UniFrac was utilized to enumerate the difference between the microbial structures among the snail gut microbiotas.

### Determination of safety properties of the potential microbial isolates

#### Hemolytic activity assay of the isolated microbial strains

The assay was done in accordance with Foulquié Moreno et al. [29]. Columbia agar (BD Difco) with 7% (v/v) sheep blood was used to prepare blood agar plates. Plates were inoculated with fresh bacterial culture (20 µl) subsequently to incubation for 48 h at 37 °C in 5% CO_2_. The hemolytic properties of isolated strains were assessed as clear zones surrounding the colonies. Bacterial isolates that showed gamma hemolysis (no clear zones around colonies) were selected for further analysis [30].

#### Isolated microbial strains DNase activity assay

For each bacterial isolate, a loopful of overnight culture was taken using a sterile loop (with a capacity of 20 µL) and streaked on the deoxyribonuclease (DNase) agar (BD Difco) for 48 h at 37 °C [31]. Ten ml of 1 N hydrochloric acid (HCL) were poured onto the plate. Colonies surrounded with clear zones were assessed to have DNase activity.

### Determination of the antibiotic susceptibility

The resistance capability of the potential probiotic bacteria to selected antimicrobials was tested by minimum inhibitory concentration (MIC) assay. A two-fold sequential broth dilution in Mueller Hinton broth was performed as described in Clinical and Laboratory Standards Institute (CLSI 2021) with the selected antibiotics (ciprofloxacin, ampicillin, chloramphenicol, gentamycin, tetracycline and vancomycin) concentrations varying between 0.25 and 1056 µg/ml. Plates were brought into a 37 °C incubator for 24 h. The least concentrations of the antimicrobial agents with no visible turbidity were identified as MIC. As per the criteria set by the CLSI the results were presented as sensitive (S), intermediate (I), and resistant (R).

### Estimating acid tolerance and bile salts resistance of the selected bacterial isolates

Based on the methodology proposed by Hassanzadazar et al. [32], bile salts resistance and tolerance to an acidic pH value were evaluated with a few modifications. Bacterial isolates were cultivated in MRS broth in a 5% CO_2_ for 18 h at 37 °C. Using a spectrophotometer, the bacterial inoculum was adjusted to 0.1 OD600 (10^8^ cfu/ml). Bacterial isolates tolerability to acid was evaluated by adding 0.3 ml (10% v/v) aliquots of bacterial suspension to 3 ml of MRS broth previously modified to pH 2.5 and 7 and subsequently incubated in 5% CO_2_ for 6 h at 37 °C. Samples (20 µl) were withdrawn at 0, 1.5, 3 and 6 h. Serial dilutions (10 folds) using 0.1% peptone water were prepared to calculate the viable number of bacteria by spotting on MRS agar plates then incubated in 5% CO_2_ for 24 h at 37 °C. The results obtained were evaluated through comparing each subsequent interval’s bacterial viable count (CFU) to the control (pH 7).

Bacterial isolates bile tolerance was done by adding 10% v/v (300 µl) of overnight cultures of the selected isolates into 3 ml of MRS broth incorporated with 0.7% and 0.3% w/v of bile salts. MRS broth with no bile salts was considered as control. Following incubation at 37 °C in 5% CO_2_, samples were taken at 0, 1.5, 3, 6 and 24 h. The bacterial isolates’ viable counts were determined using 10-fold serial dilutions made in 0.1% peptone saline. After spotting 10 µl of aliquot on MRS gar plates, they were incubated for 24 h at 37 °C. Results were assessed through the comparison of each subsequent interval’s bacterial viable count (CFU) at 0.3% and 0.7% bile salts to the control (without bile salts).

### Impact of varying pH values on the growth of microbial isolates

Overnight cultures of the bacterial isolates in MRS broth were incubated in 5% CO_2_ at 37 °C. Using a spectrophotometer, the inoculum was adjusted to 0.1 OD600 (10^8^ cfu/ml). MRS broth with previously adjusted pH 3, 5, 7 (control), 8 and 10 using 1 N HCl and 1 N sodium hydroxide was inoculated with bacterial suspension of the selected isolates then incubated at 37 °C in 5% CO_2_ for 24 h [33]. Absorbance (600 nm) of bacterial growth was measured.

### Evaluation of the antibacterial activity against tested reference strains

Bacterial isolates antimicrobial activity towards pathogenic bacteria was estimated according to Ebrahimi et al. [34] using agar overlay method with slight modifications. Briefly, *S. aureus* Newman, *E. coli* ATCC 8739, *S. typhi* ATCC 35,664 and *Ps. aeruginosa* PA01 were used as tested pathogenic bacteria. Overnight culture (10 µl) of each of the selected isolates were spotted on MRS agar plates and incubated in 5% CO_2_ for 24 h and at 37 °C. Ten ml of MH broth with 0.8% agar containing 10% v/v of bacterial suspension of the reference strains (10^8^ cfu/ml) were overlaid on the MRS agar plates. Plates were then incubated for 24 h at 37 °C. Colonies surrounded with clear zones were considered to have antimicrobial activity against tested bacterial strains.

### Cell surface hydrophobicity evaluation

In order to evaluate the interactions between the bacterial isolates’ cell surfaces as previously published by Dushku et al. [16] but with few modifications, overnight bacterial cultures cultivated in MRS broth in 5% CO_2_ at 37 °C were centrifugated at 6000 rpm for 12 min. Using PBS, the bacterial pellet was rinsed twice and reconstituted in PBS buffer. The OD was modified at 1.0 OD 600 (A0). A bacterial culture of 6 ml was incorporated with 2 ml of a polar solvent xylene (Thermo Fisher, US) and then placed in 5% CO_2_ at 37 °C for 1 h after vortexing at 2500 rpm for 4 min to segregate the two phases. Absorbance of the aqueous phase was determined at 600 nm (At). The hydrophobicity % was acquired with this equation: (1 - At∕A0) × 100.

### Determination of auto aggregation

Assessment of the potential probiotic isolates auto aggregation was estimated by applying the methodology reported by Huligere et al. [35]. Briefly, overnight bacterial isolates cultures cultivated in MRS broth in 5% CO_2_ at 37 °C were centrifuged at 6000 rpm for 12 min. Bacterial pellet was rinsed two times with PBS. The bacterial pellet was resuspended in PBS and the OD was adjusted at 1.0 at 600 nm (OD0). The bacterial suspensions were incubated in 5% CO_2_ for 24 h at 37 °C. Absorbance was measured at time intervals 0, 2, 4, 6 and 24 (OD_t_). To calculate the percentage of auto aggregation, we used this equation: [(1 – (OD_t_/OD0)) × 100].

### MTT assay for Estimation of the anti-proliferative effect of bacterial isolates using the Caco-2 cell line

Overnight cultures of the three isolated bacterial strains in MRS broth in a 5% CO_2_ incubator at 37 °C were carried out. To acquire CFS, the bacterial cultures were centrifuged at 6000 rpm for 13 min. Nylon syringe filter (0.22 μm) was used for filtration of CFS and Maintained at -20 °C until used. To prepare for the experiment, the bacterial isolates supernatants including the metabolites were diluted in MRS culture medium to achieve three different concentrations: 100%, 50%, 25%, 12.5%, and 6% (v/v). The Caco-2 cell line from Egyptian Holding Company for Biological Products and Vaccines (VACSERA, Egypt) was incubated in Dulbecco’s Modified Eagle Culture Medium (Sigma Aldrich) with 10% v/v fetal bovine serum (FBS) and 100 µg/ml antibiotics (penicillin, streptomycin) added. Caco-2 cells were cultured and placed in 5% CO_2_ incubator at 37 °C with humidified air using 96 well plate. To facilitate cell adhesion, trypsin-EDTA was utilized. The cytotoxic effects of CFS of bacterial strains on CaCo-2 cell lines were assessed through the following MTT assay reported by Chen et al. [36] with some adjustments. The appropriate number of Caco-2 cells were added to 96-well plates and left to incubate at 37 °C for 18–21 h. The Caco-2 cells culture media was extracted and replaced with varying dilutions of the bacterial isolates CFS. Bacterial isolates cells were cultured in a 5% CO_2_ incubator for 48 h at 37 °C. After finishing the incubation period, dead cells were gently removed with phosphate buffer saline (PBS). Each well was totally covered with MTT solution. Plates were placed in the incubator for 2–3 h to allow MTT conversion by live cells. The MTT formazan crystals solution was washed out by adding dimethyl sulfoxide (DMSO). Using an ELISA reader (Biotek 800 TS, USA), the OD was estimated at 570 nm. The absorbance corresponds to the quantity of live cells. For the negative control, cells were treated with only MRS, and for the positive control, cells were incubated with DMSO. Results were calculated as a percentage of cell viability regarding the control employing the equation (OD of test-OD of medium / OD of control-OD of medium X 100). Each microbial isolate CFS IC50 value was then determined. We performed the experiments in triplicate.

### Cytopathology assay

Caco2 cells were exposed to IC50 concentrations of the bacterial isolates’ CFS and MRS medium only to exclude the effect of the media for 48 h. Subsequent to treatment, the cells were harvested in a tube, subjected to trypsinization, washed twice with PBS (pH 7.4), and centrifuged using a Shandon Cytospin (Thermo Fisher Scientific, Waltham, Massachusetts) for 10 min. at 1300 rpm. The obtained cell pellets were positioned on positively charged glass slides and preserved in 95% ethanol for a duration of one day. Slides were stained using Hematoxylin and eosin (H & E) and immunohistochemistry for caspase 3 and Tumor necrosis factor alpha (TNF-α) subsequent to fixation and examined.

### Immunohistochemical assay

Immunostaining with caspase 3/ TNF-α monoclonal antibodies was performed on unstained sections using a standard 3-layer procedure, as previously reported [37]. The smears needed to be rehydrated using ethanol concentrations of 100%, 95% and 70%. After smears were rinsed with PBS, 200 µl of caspase 3/ TNF-α primary antibodies (Santa Cruz, USA) were diluted 1:100 and applied to each slide. The slides were then incubated in a humid chamber at 4 °C for 24 h. After rinsing in PBS, the smears were incubated with the secondary biotinylated antibody for 30 min., followed by the avidin-peroxidase complex for an additional 30 min. as directed by the manufacturer manual (Dako real envision detection kit, USA). Before being rinsed with distilled water, the samples were counterstained with Mayer’s hematoxylin for 60 s. after being incubated with diaminobenzidine for 5 min. or until a brown color formed. We performed the process at room temperature. Moreover, negative controls where PBS was used instead of the primary antibody were incorporated. Using brown cytoplasmic staining, the caspase-3/ TNF-α antigen was expressed. We used the method described by Duan et al. [38] to determine the apoptotic index.

Apoptotic index (%) = Active caspase 3 Immunopositively cells number / Total cells number of 10 high power fields x 100.

### Statistical analysis

GraphPad prism (version 10.0.0) was utilized to create graphs and perform statistical analysis. For each concentration of the CFS of the bacterial isolates, nonlinear curve graph was utilized to calculate the half maximal inhibitory concentration (IC50). The results were presented as mean ± SD. Data was analyzed statistically using two-way ANOVA, with Dunnett’s test for multiple comparisons. At *p* < 0.05, the differences were determined to be statistically significant. All the experiments were conducted in triplicates. Effect Size (η²) Range (%) < 1% Negligible, 1% − 5.9%, small, 6% − 13.9% medium, 14% − 25% large and > 25% very large.

## Results

### Snail gut microbiota analysis

Utilizing next generation sequencing (NGS) of DNA isolated from collected stool samples of three different freshwater snails *L. carinatus*, *C. bulimoides* and *H. duryi*, 670,837, reads were yielded after denoising and removal of chimers. The rarefaction curves (Fig. S1). demonstrated that species richness has reached a saturation point as shown by the Shannon diversity index (a representation of species diversity) and observed OTU (Operational Taxonomic Unit) to adequately examine the dominating contributors of the bacterial communities and compare snails’ gut microbiota data.

**Fig. 1:**
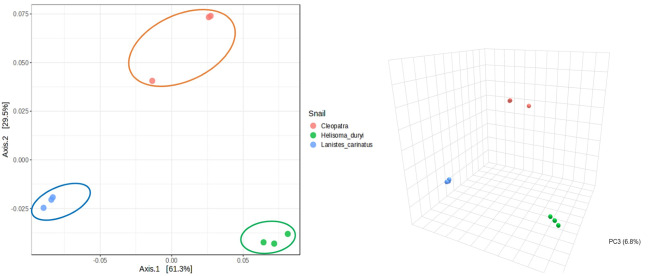
Beta diversity analysis based on principal component analysis (PCoA) of weighted UniFrac. Figure illustrates the comparison between freshwater snails’ gut microbiota at feature level using permutational multivariate analysis of variance (PERMANOVA): F-value = 22.989, R-squared = 0.88456, p value = 0.004 demonstrating significant difference between each snail species

The alpha diversity was non-significant among individual samples (*p* > 0.05) (Fig. S2). The beta diversity among snail species was analyzed by the weighted Unifrac distance metrics. Beta diversity showed clear clustering of microbial community between each snail’s gut microbiota with *p* value = 0.004, F-value = 30.191 and R-squared = 0.90961 using PERMANOVA test (Fig. 1).

**Fig. 2:**
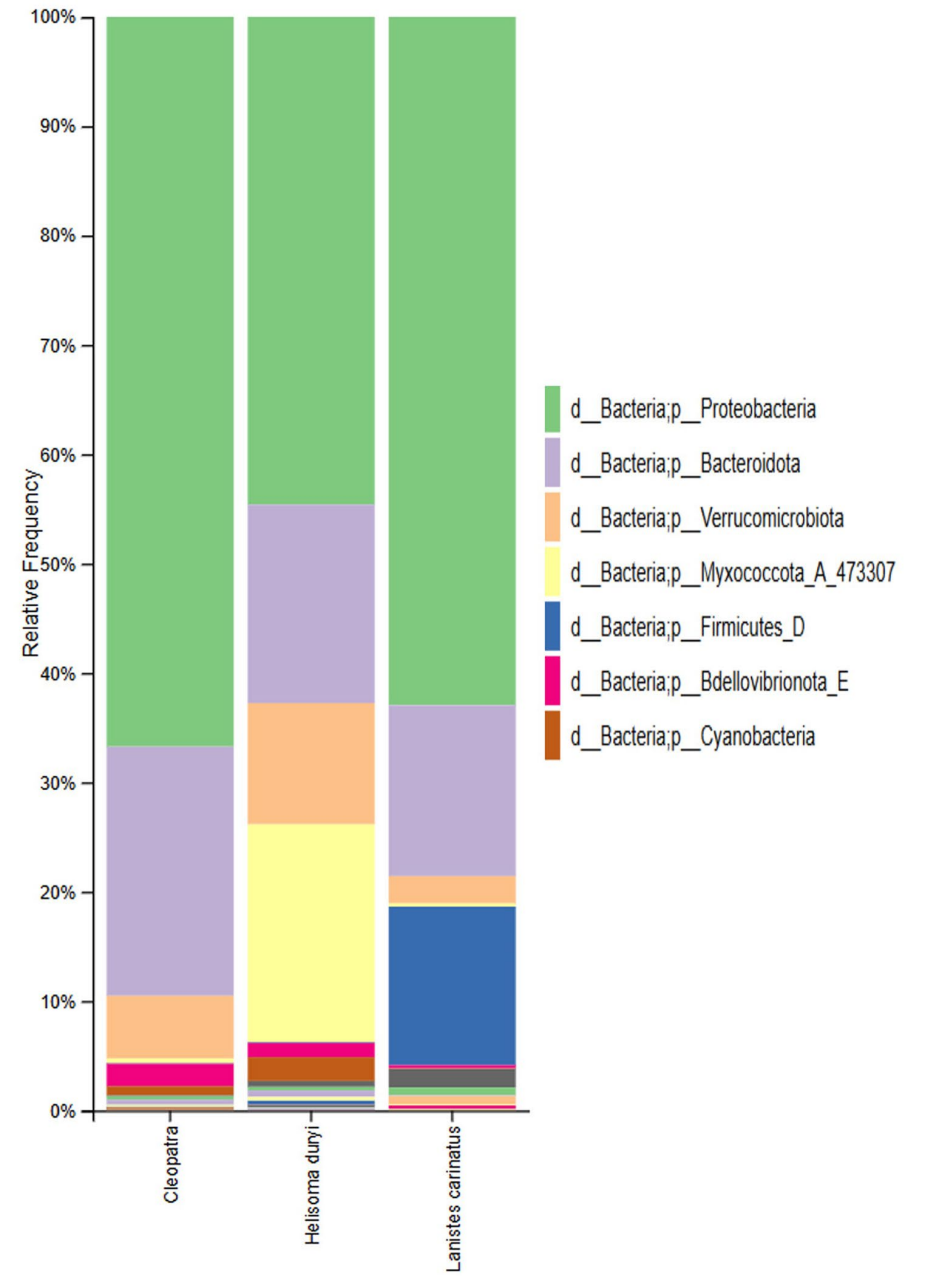
The relative abundance of the identified phyla in gut microbiota of the three freshwater snail species. The taxa demonstrate the difference in the relative bacterial abundance (%) of the most prominent phyla (Greengenes classification) among the snails’ gut microbiotas

At the phyla level, 32 phyla were identified using Qiime2 software. Two major phyla were found to dominate in all three snail species samples *Pseudomonadota* (44–70%) and *Bacteroidota* (17–20%). On the other hand, four phyla were determined to have significant differences (*p* < 0.1) among the three snails’ species. *Vernucoomicrobiota*,* Myxococcota*, and *Cyanobacteria*, were found to be more abundant in *H. duryi* with relative abundance (5–20%) than in *C. bulimoides* and *L. carinatus*. *Bacillota* phylum was found more abundant in *L. carinatus* with relative abundance of 15% while only found in < 1% in the other two snails (Fig. 2). High abundance and biodiversity of microbial taxa were observed in *H. duryi* snails compared to the other two snails.

**Fig. 3:**
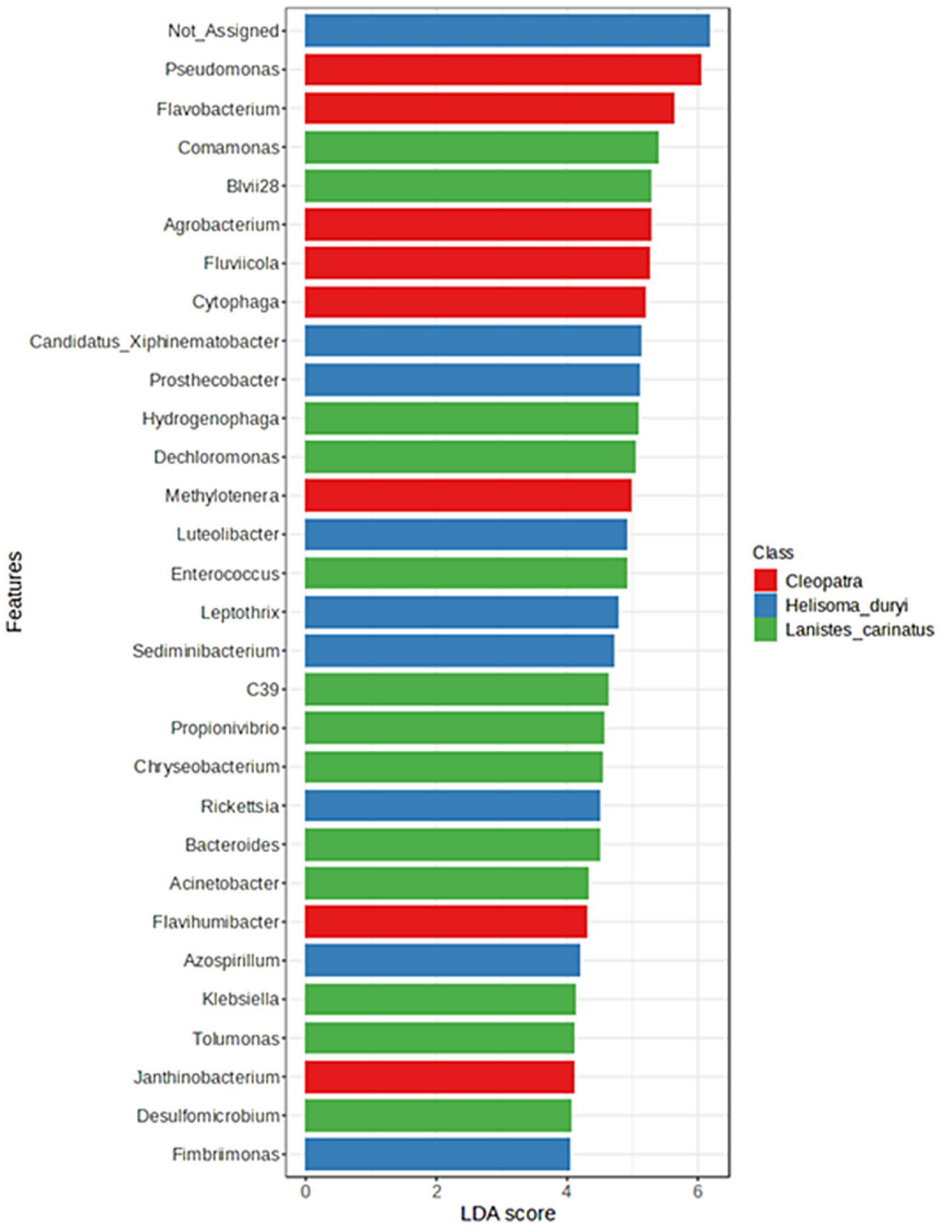
LEfSe analysis generated by microbiome analyst. The significant difference in bacterial genera among the three freshwater snails was enumerated using LEfSe (LDA score 4, *p* value < 0.05)

At the genus level, LEfSe [39] was utilized to describe the significant differences between the three snails gut microbiota (LDA score 4, *P* < 0.05). The data obtained demonstrated a statistical variation in the dominating bacterial genera abundance across all samples (Fig. 3).

On the species level, 12 bacterial species were found to be significantly variant between the three freshwater snails including high number of non-assigned bacterial species at *p* < 0.05. Among these species with beneficial benefits *Janthinobacterium lividum*, *prosthecobacter debontii*, *Zymomonas mobilis*, *Sphingomonas xenophaga* and *pseudomonas viridiflava* that were found significantly more abundant in *C. bulimoides* than the other two snails. While in *L. carinatus Brevundimonas diminuta* and *Fundidesulfovibrio putealis* were found to be more abundant and *lactobacillus johnsonii* was found in *L. carinatus* and *C. bulimoides.*

### Molecular identification of bacterial isolates

The sequences were deposited in GenBank with accession numbers (OR116116.1, KY616642.1 and OM982551.1). The BLAST analysis of the three bacterial isolates showed 97, 98 and 99% identity to *L. macroides*,* E. faecium* and *K. huakuii*, respectively which was isolated from freshwater snails *L. carinatus*, *C. bulimoides* and *H. duryi*, respectively and identified using 16 S rRNA gene sequencing (Fig. S3).

### Potential probiotic bacteria are non-hemolytic with no DNase activity

The number of potential probiotic isolates identified from each snail, which was five bacterial isolates from *L. carinatus*, four from *C. bulimoides* and six from *H. dury*. After 48 h incubation on DNase / blood agar media *L. macroides*, *K. huakuii* and *E. faecium* were the only isolates that demonstrated no clear zone around the colonies thus, confirming the non-hemolytic activity of the bacterial isolates, which indicates their safety for humans, so they were selected for further investigation (Fig. S4).

### Antimicrobial resistance profile

MIC experiment was executed to estimate the antibiotic resistance properties of the prospective probiotic bacterial isolates against six selected antibiotics. According to the findings, *E. faecium* was the only isolate sensitive to all 6 antibiotics. While *L. macroides* and *K. huakuii* were resistant to gentamycin and only *K. huakuii* was resistant to tetracycline (Table 1).

**Table 1:** Antibiotic sensitivity assessment of *L. macroides*, *K. huakuii* and *E. faecium* to six varying antibiotics via MIC determination

Bacterial isolates	MIC (µg/ml) / Susceptibility					
	Ampicillin	Chloramphenicol	Ciprofloxacin	Gentamycin	Tetracycline	Vancomycin
*L. macroides*	0.25 **S**	8 **S**	1 **S**	≥ 16 **R**	1 **S**	1 **S**
*K. huakuii*	0.25 **S**	4 **S**	1 **S**	≥ 16 **R**	≥ 16 **R**	1 **S**
*E. faecium*	0.25 **S**	8 **S**	1 **S**	1 **S**	1 **S**	1 **S**

The results were presented as sensitive (S), intermediate (I) and resistant (R)

### Tolerance of potential probiotic microbial isolates to acidic environments and bile salts

**Fig. 4:**
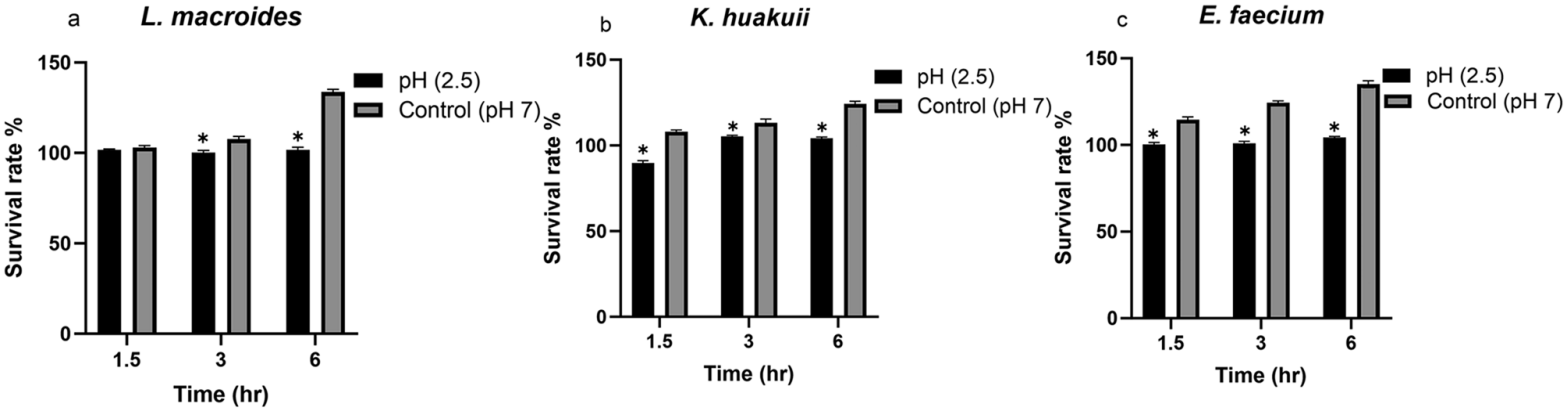
*L. macroides*, *K. huakuii* and *E. faecium* can tolerate acidic conditions. (**a**) The survival rate % of *L. macroides* in pH 2.5 and 7 (control) after 1.5, 3, and 6 h of incubation. (**b**) The survival rate % of *K. huakuii* in pH 2.5 and 7 (control) after 1.5, 3, and 6 h of incubation. (**c**) The survival rate % of *E. faecium* in pH 2.5 and 7 (control) after 1.5, 3, and 6 h of incubation. There was a significant difference between the bacterial isolates and the control (*p* < 0.05) at each time interval except for *L. macroides* which was after 3 h. Effect sizes for acidic pH: *L. macroides* (33.24%), *E. faecium* (77.7%) and *K. huakuii* (54.42%). Data shown are mean ± SD of triplicate values of independent experiments. * Values represent significant difference (*p* < 0.05)

The survival in the stomach and colon of the host is a significant trait of a probiotic bacterium. *L. macroides*, *K. huakuii* and *E. faecium* distinguished ability to withstand the acidic pH value (pH 2.5) after 1.5, 3 and 6 hr of incubation in 5% CO_2_ at 37°C demonstrating no substantial reduction in the bacterial count (> 97%). Statistical variation in comparison to the control was observed as acidic pH exerted the strongest effect on *E. faecium* survival (η² = 77.7%, *p* < 0.0001). For *K. huakuii*, pH (η² = 54.4%, *p* < 0.0001) showed high effects. In contrast, *L. macroides* displayed large effects (η² = 33.2%, *p* < 0.0001) at 3 hr (Fig. 4). Full statistics in (Table S1). For each bacterial isolate, survival rates at different exposure times (1.5 hr, 3 hr, 6 hr) were compared as shown in (Table S2). Our analysis revealed that while numerical reductions in *L. macroides* survival were observed across all time intervals, these decreases were not statistically significant. This finding is a strong indicator of our isolate’ robust acid tolerance, a crucial characteristic for their probiotic potential. While *E. faecium* also showed high tolerance as a very small but statistically significant decrease was observed between 3 and 6 h, its survival rate remained high throughout the experiment, confirming its overall resilience. *K. huakuii* exhibited significant initial responses to acid stress, with notable differences between 1.5 h vs 3 h (*p* < 0.01) and 1.5 h vs 6 h (*p* < 0.05). However, after 3 h of exposure there was no significant difference between 3 h vs 6 h. This finding can be attributed to the nature of acid stress on bacterial physiology.

**Fig. 5:**
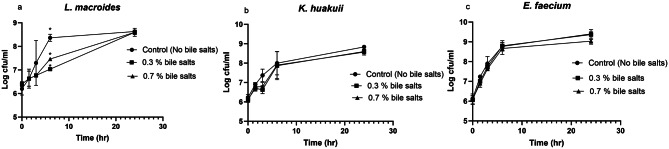
The three bacterial isolates can tolerate bile salts. (**a**) The survival of *L. macroides* in presence of bile salts concentrations (**b**) Survival of *K. huakuii* in the presence of bile salts concentrations (**c**) Survival of *E. faecium* in the presence of bile salts concentrations. Bile salts concentration had minimal effects towards all bacterial isolates (η²<2.6%). Error bars indicate standard deviation of triplicate experiments. * Values represent significant difference (*p* < 0.05)

To optimize their survival in the GIT, probiotic bacteria must be tolerant to bile salts. The degree of tolerance of *L. macroides*, *K. huakuii* and *E. faecium* to different bile salts concentrations (0.3% and 0.7%) has been explored. The results indicated that the three potential probiotic bacteria can persist at all bile salts concentrations and grow in number linearly throughout the incubation time (0–24 h). There was no significant variation between the tested bile salts concentrations and the control (MRS with no bile salts). *L. macroides* (η² = 2.60%, *p* = 0.0754) despite showing significant difference at 6 h time point only, *K. huakuii* (η² = 1.71%, *p* = 0.2521) and *E. faecium* (η² = 0.07%, *p* = 0.8159) demonstrated minimal effects to bile salts concentration (Fig. 5). Full statistics in (Table S3).

### Survival of the microbial isolates in different pH values

The effect of acidic and alkaline pHs on the bacterial growth of the three bacterial isolates were determined as shown in (Fig. S5). There was no substantial reduction in bacterial OD of the three microbial isolates at pH 3 after 24 h of incubation while at pH 5 the bacterial OD increased despite the significant difference in comparison to control (pH 7). However, at pH 8 and 10 there was no significant difference between the bacterial OD in comparison to control (pH 7) for the three microbial isolates.

### The antimicrobial activity of the isolates against pathogenic bacteria

An agar overlay experiment was used to test the antagonistic impact of the three bacterial isolates on *S. aureus*,* E. coli*,* S. typhi* and *Ps. Aeruginosa* (Fig. S6). All isolates have been shown to be antagonistic to all 4 pathogens establishing their antimicrobial activity with *K. huakuii* showing greater inhibition zones than the other two bacterial isolates (Table 2).

**Table 2:** The Inhibition of pathogenic bacteria by the three microbial isolates

Pathogenic microorganism	ZDI ± SD (mm)		
	*E. faecium*	*L. macroides*	*K. huakuii*
*Staphylococcus aureus* (Newman)	26 ± 1.4	31.3 ± 0.9	32.3 ± 0.8
*Salmonella typhimurium* (ATCC 35664)	28.6 ± 1	26 ± 0.9	32.3 ± 0.8
*Pseudomonas aeruginosa* (PA01)	26 ± 1.1	28 ± 1	29.3 ± 0.8
*Escherichia coli* (ATCC 8739)	23.3 ± 0.8	24 ± 0.8	29.5 ± 0.8

ZDI: zone diameter of inhibition; SD: standard deviation

### Auto aggregation and cell surface hydrophobicity properties

Isolated potential probiotic bacteria retain good cell adhesion properties. The three bacterial isolates (*E. faecium*, *L. macroides* and *K. huakuii*) auto aggregation percentages were increased significantly over the time intervals 2, 6 and 24 h. *E. faecium* and *L. macroides* showed maximum auto aggregation percentage (90%) while *K. huakuii* showed only 70%.

Xylene was utilized to calculate cell surface hydrophobicity of the potential probiotic bacterial isolates. *E. faecium* exhibited high hydrophobicity (> 75%) while *L*. *macroides* and *K. huakuii* exhibited 62% and 40%, respectively as shown in (Table 3).

**Table 3:** Auto aggregation and hydrophobicity of the three bacterial isolates

Bacterial isolates	Auto aggregation %			Hydrophobicity %
	after 2 h	after 6 h	after 24 h	after 24 h
*L. macroides*	15 ± 0.5	30 ± 0.5	90 ± 0.7	62 ± 0.3
*K. huakuii*	25 ± 0.6	60 ± 0.8	70 ± 0.5	40 ± 0.4
*E. faecium*	30 ± 0.5	40 ± 0.7	90 ± 0.3	75 ± 0.2

Data represented as ± standard deviation of triplicates expressed in percentage

### Cytotoxic activity of the isolated bacterial strains against Caco-2 cells

**Fig. 6:**
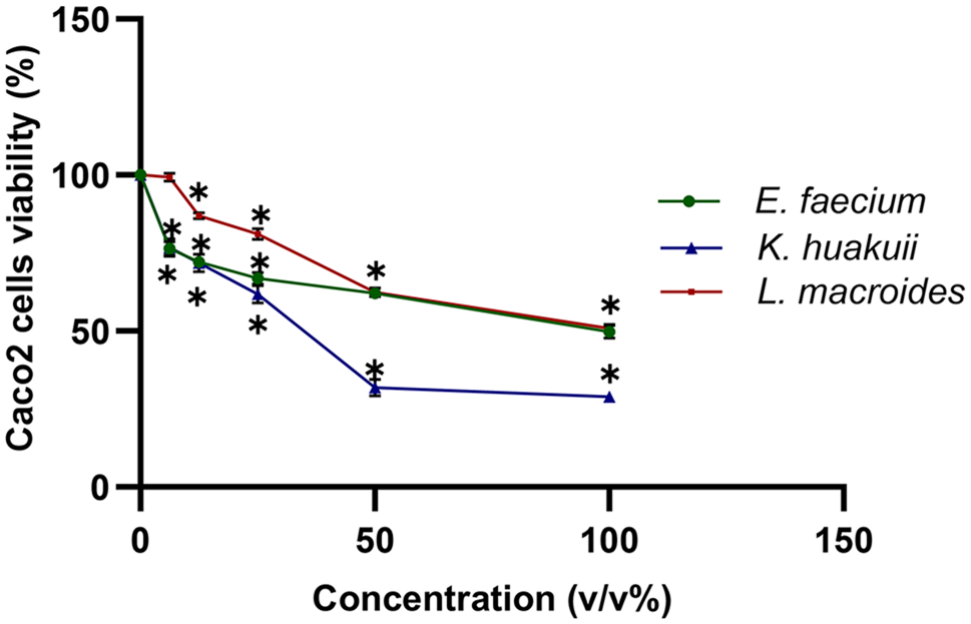
The three bacterial isolates cytotoxic activities against Caco2 cells. Caco-2 cell survival percentage of *L. macroides*, *K. huakuii* and *E. faecium* cell free supernatants at different concentrations compared to the control of equivalent concentrations (sterile MRS broth). Error bars indicate standard deviation of triplicate experiments * Values represent significant difference (*p* < 0.05)

MTT cell viability analysis was adopted to assess the cytotoxicity activity of *L. macroides*, *K. huakuii* and *E. faecium* against the human colon Caco-2 cell line. The cytotoxic effects of each bacterial isolate CFS in varied dilutions (v/v %) after 48 h incubation (Fig. 6). On the Caco-2 cells, *K*. *huakuii* showed the most anticancer activity with IC50 value of 30% v/v, followed by *L. macroides* and *E. faecium* with IC50 90% for both bacterial isolates.

### Morphological apoptosis assessment

**Fig. 7:**
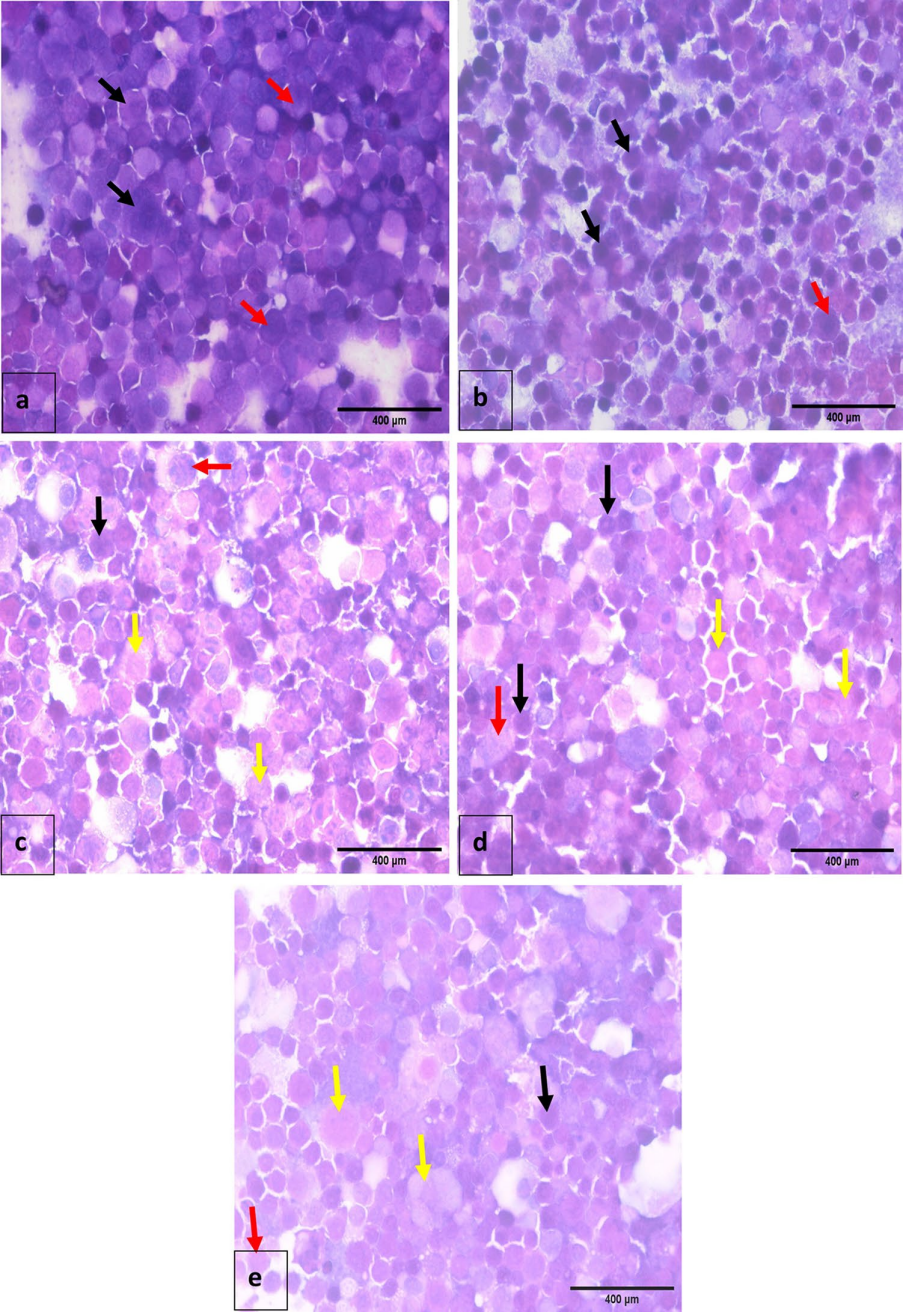
Cytospin smear stained by H & E (400×): **a** and **b**: Untreated Caco-2 cells (control) and Caco-2 cells treated with MRS medium showing a large number of neoplastic cancer cells consisting of groups of epithelial cells with enlarged nuclei (black arrow) and increased nucleocytoplasmic ratio (red arrow). **c**: Caco-2 cells after treatment with IC_50_ of CFS of *L. macroides* showing moderate number of colon carcinoma cell line (black arrow) and mild number of small shrinking cells with central nuclei, with mild apoptotic (red arrow) and degenerative changes (yellow arrow). **d**: Caco-2 cells after treatment with IC_50_ of CFS of *K. huakuii* showing a mild number of colon carcinoma cell line (black arrow) and a large number of small shrinking cells with central nuclei, large scattered apoptotic changes (red arrow) and degenerative changes (yellow arrow). **e**: Caco-2 cells after treatment with IC_50_ of CFS of *E. faecium* showing a mild number of colon carcinoma cell line (black arrow) and moderate number of apoptotic (red arrow) and degenerative changes (yellow arrow)

The appearance and properties of Caco-2 cells were affected after treatment with IC50 of CFS of the three bacterial isolates. Untreated cells stained with H & E showed neoplastic Caco-2 in the form of clusters of large cells with enlarged nuclei and high nucleocytoplasmic ratio. Similarly, Caco-2 cells treated with MRS only exhibited a high number of neoplastic cancer cells and an increased nucleocytoplasmic ratio. In contrast, Caco-2 cells exposed to IC50 concentration of *K. huakuii* exhibited more noticeable apoptotic alterations including a small cell size, nuclear chromatin condensation and disintegration, and membrane blebbing. On the other hand, medium levels of apoptotic and degenerative alterations were seen in Caco-2 cells treated with the IC50 concentrations of *L. macroides* and *E. faecium* (Fig. 7).

### Effect of IC_50_ of the three bacterial isolates on Caspases- 3 and TNF-α expression identified by immunohistochemistry

**Fig. 8:**
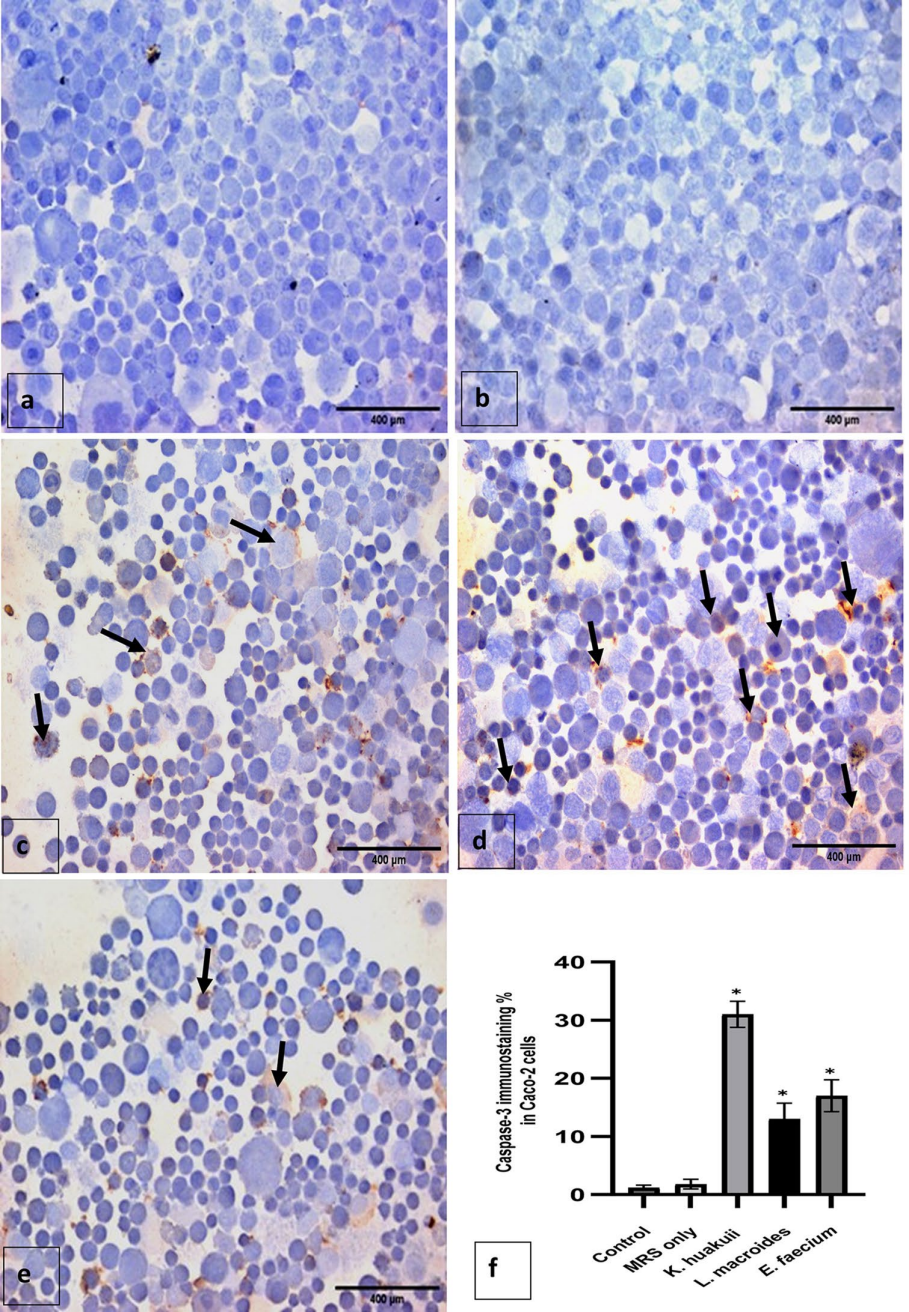
Immunohistochemical cytospin smear (caspase 3, DAB, 400×). **a**-**b**: Untreated Caco-2 (control) and treated Caco-2 cells with MRS medium showing negative staining for caspase-3. **c**: Treated Caco- 2 cells with the IC_50_ of CFS of *L. macroides* demonstrating moderate number of cells expressing caspase 3 as brownish cytoplasmic coloration in the Caco-2 with apoptotic changes (arrows). **d**: Treated Caco- 2 cells with the IC_50_ of CFS of *K. huakuii* showing a large number of cells expressing caspase 3 as brownish cytoplasmic coloration in the Caco-2 cells with apoptotic changes (arrows). **e**: Treated Caco- 2 cells with the IC_50_ of CFS of *E. faecium* a mild number of cells expressing caspase 3 as brownish cytoplasmic coloration in the Caco-2 cells with apoptotic changes (arrows). **f**: The apoptotic index of caspase 3 in Caco-2 cells treated with CFS of the three bacterial isolates. Values as presented as mean ± SD. one way ANOVA was used to compare each group with the corresponding control (untreated Caco-2 cells). * Values represent significant difference (*p* < 0.05)

There were no caspase-3 positive cells found in Caco-2 cells that weren’t treated or treated with MRS medium only. A caspase-3 was found in cells treated with CFS of the three bacterial isolates with varying degrees. We determined the apoptotic index by dividing the number of cells expressing caspase-3 by the total cell count. Caco-2 cells treated with the CFS of *K. huakuii* (30.2 ± 1.51), *L. macroides* (13.2 ± 1.4) or *E. faecium* (15 ± 1.12) were significant different in comparison to untreated or MRS medium treated Caco-2 cells (*P* < 0.05). Caco-2 cells treated with *K. huakuii* showed the highest apoptotic index followed by *E. faecium* and *L. macroides* as shown in (Fig. 8).

**Fig. 9:**
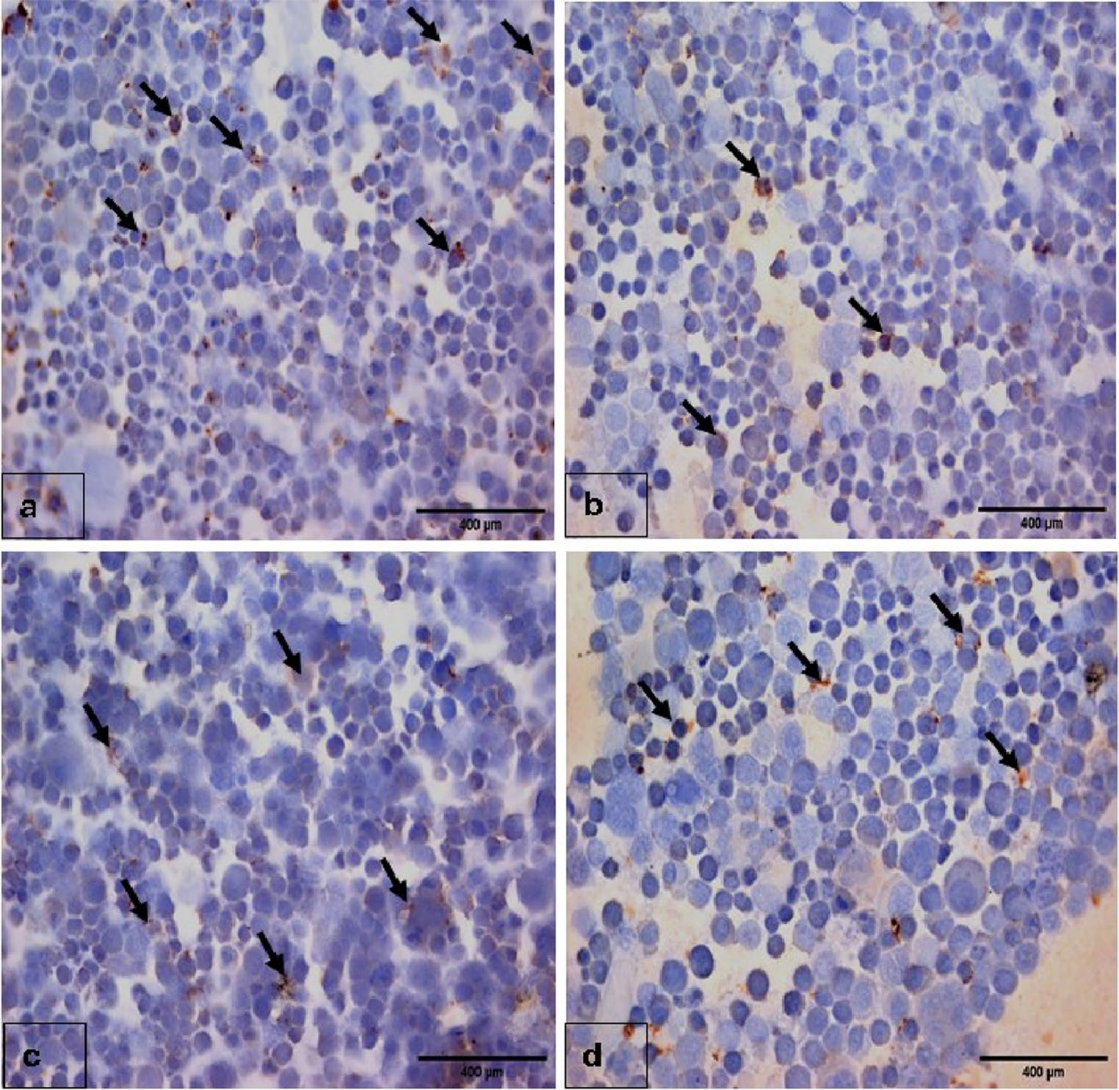
Immunohistochemical cytospin smear (TNF-α, DAB, 400×). **a**: Untreated Caco-2 (control) showing positive staining for TNF -α as brownish cytoplasmic coloration in the Caco-2 cells (arrows). **b**: Treated Caco- 2 cells with the IC_50_ of CFS of *L. macroides* demonstrating positive staining for TNF -α as brownish cytoplasmic coloration in the Caco-2 cells (arrows). **c**: Treated Caco- 2 cells with the IC_50_ of CFS of *K. huakuii* showing mild positive staining for TNF -α as brownish cytoplasmic coloration in the Caco-2 cells (arrows). **d**: Treated Caco- 2 cells with the IC_50_ of CFS of *E. faecium* showing positive staining for TNF -α as brownish cytoplasmic coloration in the Caco-2 cells (arrows)

Expression of TNF-α in untreated Caco-2 cells and treated cells with the IC50 concentrations of *L. macroides* and *E. faecium* was positive as brownish cytoplasmic coloration in cells. However, the TNF-α was mildly expressed in cells treated with IC50 of *K. huakuii* which suggest the mild anti-inflammatory activity of *K. huakuii* on Caco-2 cells while *L. macroides* and *E. faecium* possess no activity as shown in (Fig. 9).

## Discussion

Throughout history snails have been valued for their nutritive and medicinal benefits. However, microbial populations have not been extensively studied [40] and only a limited research has yielded knowledge about freshwater snails gut microbial communities [41, 42]. Most of the freshwater snails are used as bioindicators of heavy metals [43] such as *L. carinatus* which is used as bioindicator for crude oil pollution [44] and chlorpyrifos an organophosphorus pesticide [45]. *H. duryi* is also used as biological controller [46]. *L. carinatusis* was reported by El-Khayat et al. [47] to be the second most common species among the many snail species in the river Nile, followed by *C. bulimoides.*

The ability of freshwater snails to provide resilient ability for snail survival in polluted environments by absorbing or breaking down some toxins [48] through their intestinal microbiota signifies the divers potential of their gut microbiota. More intriguing is that the three selected freshwater snails are non-parasite transmitting snails which can be associated with their gut microbiota as they can generate bioactive molecules against possible pathogens and parasites or by strengthening their immunological reactions [49]. Our study species *L. carinatus*, *C. bulimoides*, and *H. duryi* are not intermediate hosts for human parasites. *C. bulimoides* and *L. carinatus* are not commonly reported as intermediate hosts for human parasitic diseases. *H. duryi* is particularly notable as it has been used as a biological control agent against *schistosomiasis* intermediate hosts due to its resistance to schistosome infection and its ability to compete with and displace the intermediate host snails [50]. This non-parasitic status is important because the bacterial communities in the snails gut microbiota reflect the normal microbiota rather than those that have been altered by parasites [41]. Moreover, they are considered as niche sources of probiotics since parasites do not undergo active life cycles, the bacterial strains that are isolated are free from microorganisms associated with living parasites [51].

Our analysis revealed significant differences in microbial diversity among the three snail species, with 32 distinct phyla identified with *H. duryi* harboring 25 phyla. *Pseudomonadota* and *Bacteroidota* dominated across all species, which partially coincide with the observation of [52, 53] who reported that the gut microbiota of *H. duyri* is derived mainly from *Pseudomonadota*,* Bacteroidota*, and *Acidobacteria*. However, we discovered in *H. duryi* gut microbiota *Myxococcota* (20%) *Vernucoomicrobiota* (10%) and *Cyanobacteria* (3%) were present at a higher percentage than in the other two snails. The presence of *Myxococcota* in the gut microbiota of *H. duryi*, which is intriguing from an ecological perspective since these bacteria are known to form biofilms and to be predators [54] which suggests they may provide them with strong immunity, especially against infectious disease.

Notably, phyla *Bacillota* dominated 15% of all relative abundance in *L. carinatus* and was scarce in the other two snail species (< 1%). *Bacillota* are known to produce spores and endure harsh conditions [55] thus, *L. carinatus* might inhabit areas that are very dynamic or stressful. Furthermore, this might contribute to the use of *L*. *carinatus* as a bioindicator for pesticide and crude oil contamination. The distinct microbial communities seen in the three species of freshwater snails demonstrate their ability to adapt to specific ecological niches.

On the family level, *Burkholderiaceae* and *Pseudomonadaceae* were the two main bacterial families among all three snail species with different percentages of abundance.

Often forming symbiotic connections with their hosts, the intestinal flora of snails helps digestion and supplies vital nutrients. Furthermore, *Hydrogenophaga* hydrogen producing capabilities was reported by Kimura and Okabe [56] which contributes in the sustainable energy applications, while *Chryseobacterium* provides promising biofertilizers [57]. The identification of *Klebsiella* is significant for its established role in the biotechnological field as a source of important chemical compunds [58]. *Acinetobacter* which is reported to contributes in many eniviromental and biotechological applications [59], and *Desulfomicrobium* plays crucial roles in bioremediation of sulfur-contaminated environments [60]. Because of its nitrogen-fixing capabilities and its ability to promote plant growth, *Azospirillum* presence is useful [61]. In addition to these biotechnological uses, identifying well-known probiotic genera like *Enterococcus*, *Lactococcus*, and the predatory bacterium *Bdellovibrio* proves the importance of freshwater snails’ gut microbiota as a niche source for beneficial bacteria.

On the species level, several bacterial species with antifungal and antimicrobial abilities were identified such as *Pseudomonas viridiflava* [62], *Janthinobacterium lividum* [63] and *Bdellovibrio bacteriovorus* [64]. In addition to bacterial species with bioremediation application as *Desulfovibrio putealis* [65], *Sphingomonas xenophaga* [66] and *Brevundimonas diminuta* [67].

Freshwater snails can be found in lakes, wetlands, ponds, and waterways, where they feed on algae, water plants, plant leaves, and many types of detritus [53]. These sources of nutrition require a diversified gut microbial population for metabolism, which aligns with the various microbial populations found in snail guts in this study. According to Madsen [68] detritus is their primary food source, followed by decomposing macrophytes, and algae for *H. duryi*. Other factors that affect the microbial population of freshwater snails is water quality and sex [42, 52].

Probiotics have been extensively investigated and used for their possible health advantages, and researchers are always seeking niche sources to identify new probiotic strains [69, 70]. Snail gut is one such unorthodox source that has received recent interests [14, 16]. In this research, the probiotic characteristic of three microbial strains isolated from the fecal microbiota community of three freshwater snails *L. carinatus*, *C. bulimoides*and *H. duryi* and identified as *E. faecium*, *L. macroides* and *K. huakuii*, respectively was confirmed by primary evaluations, which included evaluation for low pH and high bile salt tolerance, antagonistic activity against pathogens, and antibiotic susceptibility. One of the primary probiotic safety properties investigated in the three bacterial isolates is hemolytic and DNase activity where all three bacteria demonstrated no activity in both assays, an observation which is in agreement with those of Choeisoongnern et al. [71] and Zeng et al. [72]. Furthermore, resistance to antibiotics is a vital concern in the usage of probiotics. Resistance genes can be transmitted horizontally to other species, causing wide spread antibiotics resistance [73]. *E. faecium* was susceptible to all tested antibiotics which align with Kim et al. [74]. *L. macroides* was resistant to gentamycin while Devi et al. [75] reported its susceptibility. *K. huakuii* was resistant to gentamicin and tetracycline.

In our study, *E. faecium*, *L. macroides* and *K. huakuii* exhibited a noticeable tolerance to acidic pH (pH 2.5) for 6 h with survival percentage more than 97% enabling its survival through the stomach. *E. faecium* and *L. macroides* tolerance to 0.7% and 0.3% v/v of bile salts for 24 h was non-significant when compared to MRS without bile salts. These findings coincide with those of Mani et al. [76] and Shi et al. [77]. Contrary, *K. huakuii* displayed significant difference after 6 h of incubation in the presence of both bile salts concentrations but after 24 h there was no significant variation in comparison to the control (MRS without bile salts). This survival capability is an essential characteristic for probiotic bacteria, as it ensures their viability and colonization of the gut environment.

Our results revealed the impact of acidic and alkaline environments (pH 3, 5, 7, 8 and 10) on the growth of *E. faecium*,* L. macroides* and *K. huakuii* which was evaluated by measuring the bacterial optical density (600 nm) after 0, 1, 3 and 6 h. The survival percentage of the three bacterial isolates remained above 98% in pH 3 and 5. Furthermore, in pH 8 and 10 *E. faecium*, *L. macroides* and *K. huakuii* survival percentage was non-significant in comparison to the control (pH 7) as stated by Mani et al. [76]. The results demonstrate the ability of potential probiotic bacteria to maintain high cell viability in wide range of pH conditions which is vital for the sustainability and colonization of the probiotic bacteria throughout the digestive tract to exert their beneficial effects.

We found that *E. faecium*, *L. macroides* and *K. huakuii* displayed antibacterial properties against *Staph. aureus*,* E. coli*,* S. typhi*, and *Ps. aeruginosa* with *K. huakuii* exhibiting greater zones of inhibition against the tested bacteria. Several studies reported that *E. faecium*, *L. macroides* and *K. huakuii* demonstrated antimicrobial activity towards various pathogens [78–80]. This antimicrobial activity promotes a healthy gut microbiota by suppressing the proliferation of pathogenic microbes and potentially lowering the incidence of gastrointestinal illnesses [81].

We tested the cytotoxicity of *E. faecium*, *L. macroides* and *K. huakuii* in vitro using caco2 cell line. The findings of our MTT assay, which demonstrate that *K. huakuii* possesses the lowest IC50 compared to *L. macroides* and *E. faecium*, are confirmed with the observation of the morphological changes in Caco-2 apoptotic cells and the immunohistochemistry analysis. Caspase-3 is one of the pro-apoptotic caspases [82]. It is frequently utilized as an indicator of cancer treatment efficacy as it’s responsible for nuclear alterations in apoptosis and essential components of the apoptotic cascade [83].

Apoptosis has been the primary target treatment in cancer [84] as it eliminates cancer cells effectively without inducing the inflammatory response associated with necrotic cell death [85]. Our results revealed that cell death occurs through apoptotic mechanisms rather than necrosis, as evidenced by the high levels of this enzyme in the Caco-2 cell treated with CFS of the three bacterial isolates with *K. huakuii* being the highest with an apoptotic index of 30.20 ± 1.51. Although cells treated with *L. macroides* (13.20 ± 1.4) and *E. faecium* (15.00 ± 1.12) had moderate caspase-3 levels indicates that these isolates can also induce apoptosis, but to a lesser extent compared to *K. huakuii* which in agreement with Pourmollaei et al. [86] who reported the anticancer abilities of *E. faecium* cell-free supernatant.

TNF -α is a proinflammatory cytokine frequently associated in the promotion of cancer growth [87]. Among the three bacterial isolates only *K. huakuii* exhibited mild inhibition of the TNF-α expression in Caco-2 cells which suggest it’s anti-inflammatory effects alongside its anticancer effects.

Intestinal colonization of probiotics in the digestive system is due to auto aggregation and hydrophobicity, and it is a critical factor influencing probiotic assessment. The auto aggregation characteristic allows bacteria from the same species to attach to the colon mucosa [88]. *E. faecium*, *L. macroides* and *K. huakuii* showed cell-surface hydrophobicity with values 75, 62 and 40%, respectively. On the other hand, *E. faecium* and *L. macroides* demonstrated high auto aggregation (90%) and *K. huakuii* 70%. These findings were found to be aligned with those of Abedini et al. [75] and Devi et al. [89].

The demonstrated probiotic features of our bacterial isolates show significant potential in therapeutic applications. Their tolerance to acid and bile salts enables their use as oral probiotics in functional fermented foods and biopharmaceuticals [90]. Moreover, their antimicrobial activity offers an alternative to antibiotics or prophylactics against gastrointestinal pathogens promoting gut health, suppressing infections, and regulating dysbiosis [91]. Specific bacterial strains are reported as adjunct cancer therapies [92]. Recent clinical study has demonstrated that probiotics with anticancer properties can enhance chemotherapy efficacy while reducing treatment-related toxicity [93].

## Conclusion

In conclusion, our study provides valuable insights into the gut microbiota of freshwater snails, that is formed by their diverse diets and habitats and presents a rich source of novel bacteria with potential applications in health, bioremediation, and other applications. Furthermore, probiotic potential and anticancer activity of *E. faecium*, *L. macroides*, and *K. huakuii* isolated from freshwater snails were investigated in vitro. While *E. faecium* has been extensively studied, *L. macroides* and *K. huakuii* represented promising but underexplored candidates. However, further research, including in-vivo studies, is needed to fully realize their potential and ensure their safe and effective use.

## Limitations and future directions

Although our in vitro findings are encouraging, in vivo research is required to confirm probiotic effectiveness, including influence on host immunity and microbiome modification. Further studies with larger sample size, in addition to identification of compounds responsible for their anticancer activities and genome sequencing of isolates is to be considered.

## Supplementary Information

Below is the link to the electronic supplementary material.

Supplementary Material 1

## Data Availability

This study’s conclusions are supported by the 16S rRNA sequencing data that has been uploaded to NCBI (BioProject: [PRJNA1186734] (https://www.ncbi.nlm.nih.gov/bioproject/PRJNA1186734)).

## Abbreviations

ANOVA: Analysis of variance
ATCC: American Type Culture Collection
BLAST: Basic Local Alignment Search Tool
Caco-2: Human colorectal adenocarcinoma cell line
CFU: Colony forming unit
CFS: Cell free supernatants
*C. bulimoides*: *Cleopatra bulimoides*
CLSI: Clinical and Laboratory Standards Institute
CO₂: Carbon dioxide
DMSO: Dimethyl sulfoxide
DNA: Deoxyribonucleic acid
DNase: Deoxyribonucleases
*E. coli*: *Escherichia coli*
EDTA: Ethylenediaminetetraacetic acid
*E. faecium*: *Enterococcus faecium*
FBS: Fetal bovine serum
GIT: Gastrointestinal tract
HCL: Hydrochloric Acid
*H. duryi*: *Helisoma duryi*
IC₅₀: Half-maximal inhibitory concentration
*K. huakuii*: *Kurthia huakuii*
*L. carinatus*: *Lanistes carinatus*
LEfSe: Linear discriminant analysis effect size
*L. macroides*: *Lysinibacillus macrolides*
MIC: Minimum inhibitory concentration
MRS: Man Rogosa and Sharpe
MTT: 3-(4,5-Dimethylthiazol-2-yl)-2,5-diphenyltetrazolium bromide
NCBI: National Center for Biotechnology Information
OD: Optical density
PBS: Phosphate buffer saline
PCoA: Principal coordinates analysis
PCR: Polymerase chain reaction
PERMANOVA: Permutational multivariate analysis of variance
*Ps. aeruginosa*: *Pseudomonas aeruginosa*
QIIME: Quantitative Insights Into Microbial Ecology
*S. aureus*: *Staphylococcus aureus*
*S. typhi*: *Salmonella typhi*
TNF-α: Tumor necrosis factor-alpha
UV: Ultraviolet
