# Supplementary material for: Probiotic, antimicrobial and anticancer properties of Lysinibacillus macroides, Kurthia huakuii, and Enterococcus faecium isolated from freshwater snail gut microbiota
Source: BMC Biotechnol. 2025 Sep 3;25:98. doi: 10.1186/s12896-025-01038-5 (PMC12406508; doi:10.1186/s12896-025-01038-5)
Supplement: Supplementary file 1 — Supplementary Material 1 [file 12896_2025_1038_MOESM1_ESM.docx]

**Supplementary material**

Article title: Probiotic, antimicrobial and anticancer properties of *Lysinibacillus macroides*, *Kurthia huakuii*, and *Enterococcus faecium* isolated from freshwater snail gut microbiota

Journal name: BMC Biotechnology

Reham Alaa Eldin Shaker^1^, Rasha A. Hashem^1^, Mariam Hassan^1,2^, Amina M. Ibrahim^3^, Yasser M. Ragab^1,4^, Rania Abdelmonem Khattab^1^

^1^ Department of Microbiology and Immunology, Faculty of Pharmacy, Cairo University, Kasr Al-Aini 11562, Cairo, Egypt

^2^ Department of Microbiology and Immunology, Faculty of Pharmacy, Galala University, New Galala City, Suez 43511, Egypt

^3^ Department of Medical Malacology, Theodor Bilharz Research Institute, Corniche El-Nile St., Imbaba, Giza, 12411, Egypt

^4^ Department of Microbiology and Immunology, Faculty of Pharmacy, Sinai University (EL-Arish Branch), 16020, Sinai, Egypt

**Corresponding author**:

Reham Alaa Eldin Shaker

Teaching assistant of Microbiology and Immunology, Faculty of Pharmacy, Cairo University, Kasr Al-Aini 11562, Cairo, Egypt.

E-mail address: [riham.gamil@pharma.cu.edu.eg](mailto:riham.gamil@pharma.cu.edu.eg)

**Fig. S1:** Rarefaction curves of the three freshwater snail stools sequenced samples. a. observed OTU (Operational Taxonomic Unit) metrics. b. Shannon metrics (index representing the diversity within samples).

**Fig. S2:** Alpha diversity analysis of the three freshwater snails. The results demonstrate no significant difference between each snail group sample with p-value: 0.79974; [ANOVA] F-value: 0.23201

**Fig. S3:** PCR amplification of the 16S rRNA gene. A photograph of 0.8 % (wt/vol) agarose gel showing the detected PCR products on gel electrophoresis for amplified segments of 16S rRNA gene. Lane 1: 1Kbp ladder for PCR product size determination and the expected size is ~1.5 kb, lane 2-4: amplicons of 16S rRNA gene by bacterial isolates.

**Fig. S4**: Hemolytic and DNase activity tests demonstrating negative results for the three bacterial isolates. a-c: Blood agar plates of K. huakuii, E. faecium and L. macroides, respectively showing gamma-hemolysis (no hemolysis). d-f: DNase agar plates of K. huakuii, E. faecium and L. macroides showing no clear zones around colonies indicating absence of deoxyribonuclease enzyme activity.

**Fig. S5**: Effect of different pHs on the bacterial growth of the microbial isolates. The bacteria growth OD (600nm) of *K. huakuii*, *L. macroides* and *E. faecium* in pH 3, 5, 7 (control), 8 and 10 after 0 and 24 hr of incubation. Error bars indicate standard deviation of triplicate experiments. * Values represent significant difference (*p* < 0.05)

**Fig. S6**: The antimicrobial activity of K. huakuii, L. macroides and E. faecium against four pathogenic bacteria used as indicators demonstrated using agar overlay method. a-c represents the zones of inhibition formed around K. huakuii, L. macroides and E. faecium, respectively against Escherichia coli. d-g represents the zones of inhibition formed around K. huakuii, L. macroides and E. faecium, respectively against Staphylococcus aureus. h-j represents the zones of inhibition formed around K. huakuii, L. macroides and E. faecium, respectively against Salmonella typhimurium. k-m represents the zones of inhibition formed around K. huakuii, L. macroides and E. faecium, respectively against Pseudomonas aeruginosa.

g

a

**Table S1:** Statistical analysis of the three bacterial isolates acid tolerance at each time interval against control

| **Bacterium** |  | **Time (hr)** | **Mean Diff.** | **95% CI** | **Cohen’s d** | **p- value** | **Sig.** |
| --- | --- | --- | --- | --- | --- | --- | --- |
| ***K. huakuii*** |  | 1.5 | -18.33 | -22.90 to -13.76 | **-14.19** | 0.0005 | *** |
|  |  | 3 | -8.000 | -15.85 to -0.152 | **-5.24** | 0.0480 | * |
|  |  | 6 | -20.00 | -25.28 to -14.72 | **-17.32** | 0.0018 | ** |
| ***L. macrolides*** |  | 1.5 | -1.333 | -3.382 to 0.7152 | **-1.63** | 0.1336 | ns |
|  |  | 3 | -7.333 | -10.50 to -4.172 | **-5.41** | 0.0034 | ** |
|  |  | 6 | -32.00 | -35.46 to -28.54 | **-20.96** | <0.0001 | **** |
| ***E. faecium*** |  | 1.5 | -14.33 | -18.88 to -9.782 | **-10.58** | 0.0009 | *** |
|  |  | 3 | -23.33 | -26.85 to -19.82 | **-21.58** | <0.0001 | **** |
|  |  | 6 | -30.67 | -38.13 to -23.20 | **-20.82** | 0.0020 | ** |

Effect sizes (Cohen’s d) calculated as d=Mean Diff.SE×(N1+N2)/2*d*=SE×(*N*1+*N*2)/2Mean Diff. **Cohen’s d**: Negative values indicate inhibition; |d| ≥ 0.8 = large effect. **Sig.**: **** (p < 0.0001), *** (p < 0.001), ** (p< 0.01), * (p < 0.05), ns (not significant)

**Table S2:** Pairwise comparisons of survival rates across time intervals for each bacterial isolate

| Bacterium | Time Comparison | Mean Difference | 95% CI of Difference | *p*-value | Significance |
| --- | --- | --- | --- | --- | --- |
| *L. macroides* | 1.5 hr vs. 3 hr | 1.333 | -1.432 to 4.098 | >0.05 | ns |
|  | 1.5 hr vs. 6 hr | 0.000 | -2.765 to 2.765 | >0.05 | ns |
|  | 3 hr vs. 6 hr | -1.333 | -4.098 to 1.432 | >0.05 | ns |
| *E. faecium* | 1.5 hr vs. 3 hr | -0.667 | -9.780 to 8.446 | >0.05 | ns |
|  | 1.5 hr vs. 6 hr | -4.000 | -11.53 to 3.582 | >0.05 | ns |
|  | 3 hr vs. 6 hr | -3.333 | -5.861 to -0.8059 | <0.05 | * |
| *K. huakuii* | 1.5 hr vs. 3 hr | -15.67 | -20.72 to -10.61 | <0.01 | ** |
|  | 1.5 hr vs. 6 hr | -14.67 | -21.35 to -7.980 | <0.05 | * |
|  | 3 hr vs. 6 hr | 1.000 | -3.378 to 5.378 | >0.05 | ns |

**Sig.**: **** (p < 0.0001), *** (p < 0.001), ** (p< 0.01), * (p < 0.05), ns (not significant)

**Table S3:** Statistical analysis of the three bacterial isolates bile salts tolerance at each time interval against control

| **Bacterium** | **Time (hr)** | **Comparison** | **Mean Diff.** | **SE of diff.** | **Cohen’s d** | **p-value** | **Sig.** |
| --- | --- | --- | --- | --- | --- | --- | --- |
| ***K. huakuii*** |  | Control vs. 0.7% | 0.03333 | 0.1764 | **0.14** | 0.9744 | ns |
|  | 1.5 | Control vs. 0.3% | 0.2333 | 0.06667 | **2.55** | 0.0636 | *ns* |
|  |  | Control vs. 0.7% | 0.3333 | 0.1202 | **2.04** | 0.1433 | ns |
|  | 3 | Control vs. 0.3% | 0.1667 | 0.2186 | **0.56** | 0.7046 | ns |
|  |  | Control vs. 0.7% | 0.2667 | 0.2108 | **0.93** | 0.4753 | ns |
|  | 6 | Control vs. 0.3% | -0.03333 | 0.1944 | **-0.12** | 0.9788 | ns |
|  |  | Control vs. 0.7% | 0.1000 | 0.2134 | **0.34** | 0.8597 | ns |
|  | 24 | Control vs. 0.3% | 0.03333 | 0.1563 | **0.15** | 0.9677 | ns |
|  |  | Control vs. 0.7% | 0.3667 | 0.1054 | **2.53** | 0.0634 | *ns* |
| ***E. faecium*** |  | Control vs. 0.7% | 0.1333 | 0.1054 | **0.92** | 0.4325 | ns |
|  | 1.5 | Control vs. 0.3% | 0.2000 | 0.08165 | **1.76** | 0.1166 | ns |
|  |  | Control vs. 0.7% | 0.1333 | 0.1333 | **0.72** | 0.5704 | ns |
|  | 3 | Control vs. 0.3% | 0.7333 | 0.2211 | **2.40** | 0.0614 | *ns* |
|  |  | Control vs. 0.7% | 0.5667 | 0.2186 | **1.86** | 0.1179 | ns |
|  | 6 | Control vs. 0.3% | 0.1333 | 0.5457 | **0.17** | 0.9578 | ns |
|  |  | Control vs. 0.7% | 0.1000 | 0.5323 | **0.13** | 0.9747 | ns |
|  | 24 | Control vs. 0.3% | 0.2333 | 0.06667 | **2.55** | 0.0786 | *ns* |
|  |  | Control vs. 0.7% | 0.2667 | 0.09428 | **2.04** | 0.1258 | ns |
| ***L. macroides*** |  | Control vs. 0.7% | -0.2000 | 0.1633 | **-0.89** | 0.4735 | ns |
|  | 1.5 | Control vs. 0.3% | 0.000 | 0.2625 | **0.00** | >0.9999 | ns |
|  |  | Control vs. 0.7% | 0.000 | 0.2981 | **0.00** | >0.9999 | ns |
|  | 3 | Control vs. 0.3% | 0.5333 | 0.5518 | **0.70** | 0.6084 | ns |
|  |  | Control vs. 0.7% | 0.5333 | 0.5518 | **0.70** | 0.6084 | ns |
|  | 6 | Control vs. 0.3% | 1.333 | 0.09428 | **10.2** | 0.0027 | ** |
|  |  | Control vs. 0.7% | 0.9000 | 0.09428 | **6.89** | 0.0074 | ** |
|  | 24 | Control vs. 0.3% | 0.03333 | 0.06667 | **0.36** | 0.8438 | ns |
|  |  | Control vs. 0.7% | 0.03333 | 0.1054 | **0.23** | 0.9319 | ns |

Effect sizes (Cohen’s d) calculated as d=Mean Diff.SE×(N1+N2)/2*d*=SE×(*N*1+*N*2)/2Mean Diff. **Cohen’s d**: Negative values indicate inhibition; |d| ≥ 0.8 = large effect. **Sig.**: **** (p < 0.0001), *** (p < 0.001), ** (p < 0.01), * (p < 0.05), ns (not significant)
